# External Costs of Risky Health Behaviors Associated with Leading Actual Causes of Death in the U.S.: A Review of the Evidence and Implications for Future Research

**DOI:** 10.3390/ijerph7062460

**Published:** 2010-06-03

**Authors:** Armineh Zohrabian, Tomas J Philipson

**Affiliations:** 1 Division of Adult and Community Health, Centers for Disease Control and Prevention CDC, 3005 Chamblee-Tucker Rd, MS-K67, Atlanta, GA 30341, USA; 2 Harris School of Public Policy Studies, University of Chicago, 1155 E. 60th Street, Suite 112, Chicago, IL 60637, USA; E-Mail: t-philipson@uchicago.edu

**Keywords:** costs, smoking, environmental tobacco smoke, alcohol, excessive drinking, obesity, poor diet, physical inactivity

## Abstract

This paper reviews the evidence on external costs of risky behaviors in the U.S. and provides a framework for estimating them. External costs arise when a person does not bear all the costs of his or her behavior. They provide one of the strongest rationales for government interventions. Although the earlier estimates of external costs no longer have policy relevance, they demonstrated that the existence of external costs was an empirical question. We recommend that the estimates of external costs be updated as insurance structures, environments, and knowledge about these behaviors change. The general aspects of external costs may apply to countries other than the U.S. after taking into account differences in institutional, policy and epidemiological characteristics.

## 1. External Costs of Health Related Behaviors

External costs arise when a person or a group does not bear all the costs of his or her actions, thus some of the costs are borne by others or by society at large. The costs that a person bears as a consequence of his or her actions are called internal (private) costs. External costs are not considered in a person’s decision making because they are borne by others. Possible external costs of any health-related behavior primarily derive from public or private group health insurance and from events directly causing harm to others (e.g., traffic crash or secondhand cigarette smoke). External costs are relevant on the grounds of fairness and efficiency. They become an issue of fairness when someone pays unwillingly or unknowingly for the actions of others. Grounds of economic efficiency apply when paying the full costs of their own action leads to an overall higher level of efficiency by a change in personal behavior, *i.e.*, the benefits are more than the losses associated with the change, regardless of who gains or loses.

This is the first comprehensive study to examine the existing evidence on the external costs of the leading actual causes of death—smoking, poor diet/physical inactivity, and excessive alcohol consumption. Despite of the large number of studies on total costs, which do not differentiate between internal and external costs [1–9], relatively few studies estimated external costs of health related behaviors. This paper develops an analytical framework for estimating external costs and discusses current controversies related to the definition and analysis of these costs. It tries to balance the economic and the public health perspectives and generates policy questions, issues and recommendations with regard to the role of external costs in developing public health policies.

## 2. Framework for Estimating External Costs

Because risky health behaviors contribute to acute and chronic diseases which lead to high medical expenses, it seems straightforward that these behaviors also lead to an overall financial burden on those with healthier behaviors due to group health insurance programs. This, however, is more complex and requires three considerations: An analysis of cost differential between the group with risky behavior and the group without it should be conducted within a lifecycle framework. The attribution of differentials in costs to each behavior should be determined while controlling for other risky behaviors and characteristics that could contribute to this cost difference. And, finally, individual contributions to health care financing and other aspects of possible financial programs through Social Security, life insurance, or sickness benefits should be included in estimation of external costs.

If a risky behavior leads to premature death, there are competing financial influences. On the one hand, higher health care costs may be imposed in the short run, but on the other hand, the society may spend less additional resources for two possible reasons: Health care expenditures increase dramatically with age and those expenses are more heavily subsidized for older adults than for younger persons. And persons with risky health behaviors may collect Social Security and pension benefits for a shorter time than those with healthier behaviors; however, the dependents of the deceased may receive public financial support. Therefore, the net financial effect of premature death is an empirical question.

There are disputable issues related to external costs. Some may argue that because Medicare and Medicaid are entitlement programs which the society has chosen to fund, they should not be considered in the estimation of external costs. However, the willingness to fund health care for elderly and poor may differ from the willingness to pay for modifiable behaviors, such as smoking or excessive drinking.

Another disputable issue is the costs born by family members. Economists generally view the family as the decision-making unit, therefore costs to family are usually considered private. However, society may cover costs associated with developmental problems of infants and children that are attributable to maternal smoking and drinking. In addition, costs imposed on children could be viewed external. When deciding to get married an adult presumably accepts the consequences of the spouse’s risky health behaviors, but children do not choose their parents.

## 3. Methods

We searched PubMed and EconLit databases for the period 1982–2008, using both indexed (keyword) and free-text terms. Boolean logic was used to establish relationships between the concepts risky behavior and cost as well as fetal alcohol syndrome and cost. We also hand-searched the working papers of the National Bureau of Economic Research and checked references of related articles. We then screened them to identify those with empirical estimation of external costs and distinguished studies which had considered the important components of external cost estimation from those which had not.

Limitations with settings, methods and assumptions of the available studies did not allow synthesis of the findings. Therefore, the findings are presented as they appeared in the literature, followed by the amount adjusted for inflation in 2009 dollars. The main methods used in estimating external costs were: (1) non-smoking smoker construct, which is based on epidemiological observation that smokers differ systematically from non-smokers [10]. Therefore, the differentials in mortality and medical care use between smokers and nonsmokers should not be attributed to the smoking habit *per se*. The same construct should be applied to other habits. (2) Cost of illness and human capital approach, which estimates costs of medical treatment and productivity losses; and (3) Willingness to pay (WTP) and value of statistical life (VSL) approaches. WTP assesses what people are willing to pay (or accept) for relatively small changes in the risk of death. Based on WTP estimates, the economists developed VSL. To understand this concept, it helps to think in terms of risk exposure. When the risk of fatal accident is 1:100,000, statistically there is 1 death per 100,000 people. Changes in the risk level imply changes in the number of statistical lives saved. The economic value is essentially the marginal rate of substitution of wealth for risk of death.

## 4. Smoking

### 4.1. Earlier Studies

The earlier studies on external costs of smoking were primarily motivated by the question whether the society subsidized smoking. It was based on the traditional view of tobacco policy, where tobacco was treated as any other product and tobacco taxes were to be set equal to external costs it imposed on society. These studies indicated that the current external costs were lower than the taxes [11–14].

The estimated lifetime external costs were $1,000 per smoker or a net external cost of 15 cents per pack of cigarettes at a 5% discount rate in 1986 U.S. dollars, not including costs imposed on family. This adjusts to $1,958 per smoker or a net external cost of 29 cents per pack of cigarettes in 2009 U.S. dollars. If the costs of maternal smoking and smoking-related fires were included, this estimate would range from 31 to 52 cents per pack in 1986 U.S. dollars ($0.61 to $1.02 in 2009 U.S. dollars) [11,12]. The average retail price of a pack of cigarettes was about $1 per pack in 1986 U.S. dollars ($1.96 in 2009 U.S. dollars).

Viscusi [14] updated these findings [11,12]. At a 5% discount rate, with tar adjustment Viscusi’s estimate of insurance externalities were $0.32 per pack in 1993 dollars ($0.48 in 2009 U.S. dollars). He further included costs of passive smoking. The estimated highest cost to society was 41 cents per pack in 1993 dollars (61 cents in 2009 dollars). Under different assumptions and discount rates, smokers saved society 18 cents per pack (27 cents in 2009 dollars). The average retail price of a pack of cigarette at the time this study was conducted was $1.69 in 1993 dollars ($2.5 in 2009 dollars). Like the previous studies, the cost of maternal smoking was not considered external [14]. However, Evans *et al*. estimated the costs of maternal smoking to be 42 to 72 cents per pack in 1994 dollars ($0.61 to $1.04 in 2009 dollars) [15], which were almost as much or higher than the overall estimated external costs for previous studies. The average retail price of a pack of cigarette was $1.75 in 1994 dollars ($2.53 in 2009 dollars). Future earning losses of low-birth weight children due to developmental and behavioral problems were not included. Hay (1991) estimated these costs at approximately $4.10 per pack in 1990 dollars ($6.73 in 2009 dollars), which was probably an extreme value [16].

Our understanding of the epidemiology, economics and overall effects of smoking has changed since these studies were conducted, and the above estimates do not have direct policy relevance today. Moreover, a new approach in behavioral economics views tobacco consumption as different from other products, suggesting that internal costs could also play a role in taxation policy. Taxation becomes a self-control device for smokers who may want to protect their future self from their current self. In this framework, the appropriate tax levels exceed the external costs [17].

### 4.2. Environmental Tobacco Smoke (ETS)

When the above studies were conducted, the health and associated economic effects of ETS were a major point of empirical contention in the literature [18]. A 2006 Surgeon General’s Report [19] concluded that the evidence to infer a causal relationship between ETS exposure, lung cancer and cardiac diseases among nonsmokers was sufficient. ETS exposure was estimated to cause 46,000 cardiac deaths—at least 10 fold higher than lung cancer deaths; over a million episodes of childhood respiratory illnesses and middle-ear infections; and over 400 sudden infant death syndrome (SIDS) deaths annually.

A study by the U.S. Environmental Protection Agency [20] concluded that the ETS costs were substantial and that the ban on smoking in all worksites would lead to total benefits between $39 billion and $71 billion annually, equivalent of $2.45 to $4.45 per pack of cigarettes in 1993 dollars. In 2009 dollars, these benefits would be between $58 billion to $105 billion annually, equivalent of $3.64 to $6.61 per pack of cigarettes.

A summary of several economic studies on ETS found that the lowest grand total estimated costs of ETS were $14.7 billion, the average were $75.4 billion and the highest were $167 billion in 2000 U.S. dollars [21]. This adjusts to $18.31 billion, $93.9 billion, and $208 billion in 2009 dollars for the lowest, average and highest grand total estimated costs respectively. Most of the studies considered the effect of ETS on infants and children.

### 4.3. External vs. Internal (Private) Cost

Sloan *et al*. were the only study to provide a systematic accounting of how the costs of smoking were distributed among smokers and nonsmokers [21]. The comparison was for estimates that indicated where costs arose not who actually paid them. The grand total of estimated costs of smoking was $323 billion annually in 2000 U.S. dollars ($402.41 billion in 2009 dollars), of which smokers appeared to impose costs outside their own families of less than $5 billion a year ($6.23 billion in 2009 dollars). In the context of nearly $70 billion spent on cigarette purchases ($87 billion in 2009 dollars), “this appears to be an extraordinarily modest amount” [21].

In 2000 the estimated present value of private cost of smoking to a 24-year old smoker was $141,181 over the lifecycle and $32.78 per pack in 2000 dollars ($175,892 and $40.84 respectively in 2009 dollars). The costs to spouses and for infant deaths (quasi-external costs) were an estimated $23,407 over the lifecycle or $5.44 per pack ($29,162 or $6.78 respectively in 2009 dollars). The external costs were much smaller—$6,201 per 24-year-old smoker, net of federal and state cigarette excise taxes paid by smokers ($7,726 in 2009 dollars). On a per-pack basis, the external cost was $2.20 in contrast to $0.76 in excise taxes paid per pack, *i.e.*, $1.44 net of taxes ($2.74, $0.95 and $1.79 in 2009 dollars respectively). In the year 2000, the average retail price of a pack of cigarettes was $3.12 ($3.89 in 2009 dollars).

In later studies the estimates of private mortality costs ranged from about $25 per pack (15 percent or higher discount rates) to an estimated $222 per pack for men and $94 per pack for women (3 percent discount rate) in 2006 dollars [22]. If adjusted to 2009 dollars, these costs would range from about $27 to $236 per pack for men and $100 per pack for women. The average retail price of a pack of cigarette in 2006 was $3.93 ($4.18 in 2009 dollars). Another estimate of private mortality costs was around $35 per pack (3 percent discount rate) in 2002 U.S. dollars ($37 in 2009 U.S. dollars) [23]. Differences in these private mortality cost estimates were mostly because of differences in the assumptions of the models.

Overall, estimates of external costs of smoking depended greatly on the choice of discount rate, on inclusion of costs of ETS-related deaths and maternal smoking, and on the value of statistical life used.

## 5. Physical Inactivity and Obesity

An early study related to externalities of physical inactivity [24] estimated that the lifetime subsidy to a person with a sedentary lifestyle was under $2,000 in 1986 dollars ($3,915 in 2009 dollars), more than the current estimate of $1,000 for external costs of smoking ($1,958 in 2009 dollars) [12], but less than the external costs for heavy drinking of $3,200 ($6,264 in 2009 dollars), even when excluding costs for crime and traffic accidents [12]. The results were most sensitive to the assumption about the effects of inactivity on mortality. If the effect was assumed to be large, sedentary persons paid much more in Social Security taxes than they lived to collect in pensions and nursing home care. If sedentary lifestyle had no effect on mortality, costs for medical care and sick leave for sedentary persons were subsidized by others.

Bhattacharya and Sood [25] estimated the loss of social welfare from obesity to be about $150 per capita of the insured population in 1998 dollars ($197 in 2009 dollars). Obesity could have external effects through health insurance coverage only if premium setting ignored obesity. This social welfare loss occurred only when two conditions existed. First, persons of normal weight in the pooled-risk insurance plan paid part of the expenditures for the people who had health problems because of excess weight; and second, being in a pooled-risk insurance plan had an impact on people’s eating and physical activity behaviors. Otherwise, it would be a case of mere payment transfer from persons of normal weight to persons with excess weight. This study had assumed that the second condition was true; however, currently available studies do not provide conclusive evidence about it [26,27]. Some studies estimated the annual obesity-attributable medical care expenditures that were paid by public health insurances [28,29], but they did not account for the payments the obese persons made for health insurance or for their tax contributions, and did not control for other risky health behaviors.

A new aspect in this area is the issue of positive externalities of obesity, *i.e.*, the non-obese may benefit from the obesity epidemic due to induced innovation in biomedical research and medicine [30]. First, biomedical researchers are quite responsive to changes in disease prevalence in the population. Private markets reward this responsiveness with profits for pharmaceutical firms, and the National Institutes of Health rewards this responsiveness with grant funding to universities and medical schools. Second, non-obese persons who suffer from conditions that are more common among the obese also benefit from innovations induced by the obesity epidemic [31].

Another new area of research is the immediate and long-term impact of maternal obesity on life and health course of the child [32], which could amount to substantial external costs for the offspring. Medical evidence of the link between fetal environment and neonatal outcome is growing, with likely influence on future adult morbidities.

## 6. Excessive Alcohol Use

Several studies provided strong evidence of high external costs associated with alcohol abuse [11,12,33–42]. The lifetime external costs of heavy drinking were estimated to be $42,000 in 1986 U.S. dollars ($82,213 in 2009 U.S. dollars), of which more than 90% was due to drunk driving, crime, and property damage [11,12]. The estimated $17.6 billion of external costs of death and injury due to drunk driving were almost 4 times the estimated $4.7 billion of penalties paid by persons convicted of drunk driving in 1986 ($34.45 billion and $9.2 billion in 2009 U.S. dollars respectively) [35]. Current alcohol taxes were found to be too low. Stricter drunk driving laws and information provisions would reduce externalities, which at the time were estimated to be $60.16 per occasion of drunk driving in 1986 U.S. dollars ($117.76 in 2009 U.S. dollars), while average penalties paid by drunk driver were only $16.15 ($31.61 in 2009 U.S. dollars) [35,36]. 13.8% of alcohol-attributable crash costs were external, and the crash costs per drink were estimated to be $0.42 in 1995 U.S. dollars ($0.56 in 2009 U.S. dollars), which included costs of pain, suffering and lost quality of life in addition to the medical, travel, property damage, productivity losses and administrative costs [37]. The estimates of crime costs ranged from $25.9 billion in 1992 U.S. dollars [38] to $99.6 billion in 1999 dollars [39]. This adjusts to a range from $40.8 billion to $128.3 billion in 2009 U.S. dollars for crime costs.

Another source of external costs is drinking during pregnancy which can lead to fetal alcohol spectrum disorders (FASD). The lifetime total costs per individual with FAS were estimated to be $2 and $2.9 million in 2002 dollars [40–42]. If discounted, these costs were $931,742 and $1,466,875 respectively in 2002 dollars [42]. After adjusting for inflation, the lifetime costs per individual with FAS were an estimated $2.4 million and $3.5 million in 2009 dollars, and the discounted lifetime values were $1.1 million and $1.8 million in 2009 dollars. Costs associated with crime attributable to FASD were not included. Furthermore, external costs were not separated from these estimated total costs.

Carpenter and Dobkin [43] found that granting legal access to alcohol at age 21 led to large and immediate increases in alcohol consumption, and that a 1 percent increase in the number of days a young adult drinks results in a 0.4 percent increase in total mortality. Given that mortality due to external causes, such as car accidents, suicide, homicide, deaths with a mention of alcohol use peaked at about age 21, their results suggested that policy interventions to reduce youth drinking could have substantial public health benefits and could reduce external costs. In addition, in 2000 and 2004 excessive alcohol use was identified as a risk factor for risky sexual behavior among youth [44,45]. In 2008 drinking was suggested to be a possible risk factor for Alzheimer’s disease [46]. These could further add to the already large external costs associated with alcohol.

## 7. Conclusions

The estimates of external costs must be updated as the health insurance structures, medical practices and technologies, environments and our knowledge about the effects of these risky behaviors change. Although many of the early estimates of externalities are no longer relevant for today’s policy, they demonstrate that the existence and magnitude of external costs are empirical questions, and that the assumptions of the models may lead to qualitatively different results. External costs are most relevant for policy when people change their behaviors in response to costs, *i.e.*, paying the full costs of their actions leads to reduction in risky behaviors. For smoking and alcohol consumption it is well documented that increasing costs through excise taxes reduces consumption [1,2]. If there is no behavioral change, then policy intervention leads to a mere transfer of payments from people without risky behavior to people with risky behavior. This serves the fairness purpose of reducing external costs, but not the efficiency purpose.

It is useful discussing how these potential external effects can be addressed most directly and why they have not been resolved yet. Aligning the private costs of risky behaviors with the social costs appear to be straight-forward to implement. However, there have been significant differences in the tax policies of smoking and alcohol. Despite of the enormous external costs of excessive drinking, excise taxes on alcoholic beverages have been little adjusted for decades, with effective rate of taxation declining over the years. On the other hand, from 2002 to 2009 cigarette tax rates were increased for 88 times by 46 states, D.C., and several US territories. In 2009 the federal tobacco tax was increased from 39 cents to $1.01, partly with the goal of reducing smoking, thus improving health. This difference may be explained by the difference in political influence of the two industries [47], but there is another important difference. For alcohol, harm and particularly externalities arise from excessive consumption. An occasional drink may even have health benefits. On the other hand, there is no safe level of smoking. The public anti-smoking sentiment appeared to have led policies and taxes on cigarettes, mostly based on growing public awareness of smoking and health [48–50]. With present public budget deficits, taxing cigarettes and alcohol has gained stronger political and public support. Taxing sugared drinks are currently proposed to help finance health-care reform in addition to expected health benefits [51,52]. However, in general, taxation may not be appropriate for obesity. First, taxes on over-consumption of food would be more appropriate than taxes on consumption, and such taxes are not feasible. Second, food is a necessity and taxing it increases the budgetary burden on poor families, because the share of income spent on food is higher for the poor than it is for the rich.

Taxation has another economic aspect. There is gain, *i.e.*, surplus, to people when the prices they pay for products they enjoy is less than their maximum willingness to pay for them. When taxation increases prices, consumer’s response will likely be reduced consumption, involving changes in private surplus. Two economic models predict this change differently. According to the model of time-inconsistent preferences, persons with risky behaviors will be better off because taxes will serve as a self-control device helping them achieve what they eventually want to do, *i.e.*, quit smoking, drinking or eating excessively. On the other hand, the standard economic model predicts that those with risky behaviors will be worse off because the policy intervention will constrain their rational choices. This loss in private surplus will be due to the fact that consumers will have to pay more for the amount of the product they still continue to consume, and they also will have to consume less of something they enjoy. Comparing changes in private surplus for these risky behaviors can be an important topic for future research. Future research should also address the substitution of one risky behavior for another when taxes were imposed to limit each behavior in isolation. This substitution might lead to higher overall external costs because the external costs of each behavior are different.

Much interest has been given to possible externalities to employers that stem from medical costs and reduced productivity due to employees’ risky health behaviors. To offset these costs, employers most likely adjust wages; this action eventually would lead individuals with risky health behaviors to partially or fully internalize their higher health care costs [53–57]. Reducing externalities would also include higher insurance premiums of co-pays for persons with risky health behaviors. Although private health insurance has started to differentiate insurance premiums on the basis of risky health behaviors, it appears unlikely that the public sector will. Most of the studies explored the question whether risky health behaviors gave rise to externalities. The other direction of causality, whether externalities associated with insurance have an effect on person’s behaviors is a relatively new area of empirical research and the results are inconclusive [26,27].

All available studies used data on adult populations with health insurance, including Medicare and Medicaid. Persons between age 18 and 64 years who were obese or who smoked or abused alcohol were more often uninsured than those who did not have these risky behaviors [58]. Being uninsured before one becomes eligible for Medicare may affect overall lifetime external costs. These costs may increase if (1) Medicare pays for severe conditions that could have been prevented or controlled if persons with risky behaviors had health insurance earlier in life and (2) the external costs when covered by health insurance were lower than the increase in Medicare expenditures later in life. On the other hand, external costs could decrease if being uninsured reduced longevity, leading to overall reduction in Medicare expenditures and pensions.

External costs provide one of the strongest economic justifications for government interventions, but there are other justifications, such as public health ethics and altruism [17,18,59–63]. Creating programs that get people pay the full costs of their behavior, providing information about health consequences of these behaviors, taxing risky habits to protect one’s future self from their current self—all of these approaches could improve health. However, maximizing health at any cost may not be the exclusive objective of policy. Trading health for money is not uncommon [64,65]. Thus, in reports of future research focusing on public intervention to reduce risky health behaviors, explicit statement of the rationales for government interventions would be useful.
